# Paraquat toxicity in different cell types of Swiss albino mice

**DOI:** 10.1038/s41598-022-08961-z

**Published:** 2022-03-21

**Authors:** Bilal Onur, Kültiğin Çavuşoğlu, Emine Yalçin, Ali Acar

**Affiliations:** 1 Department of Biology, Graduate School of Natural and Applied Sciences, Giresun University, 28200 Giresun, Turkey; 2 Department of Biology, Faculty of Science and Art, Giresun University, Giresun, Turkey; 3 Department of Medical Services and Techniques, Vocational School of Health Services, Giresun University, Giresun, Turkey

**Keywords:** Biological techniques, Molecular biology

## Abstract

In this study, toxicity caused by 50, 100 and 200 mg/kg b.w doses of Paraquat herbicide in Swiss albino mice was investigated. Body weight, liver and kidney organ weights, aspartate aminotransferase (AST) and alanine aminotransferase (ALT) enzyme activities, blood urea nitrogen (BUN) and creatinine levels, malondialdehyde (MDA) and glutathione (GSH) levels in liver and kidney, micronucleus (MN) formation in buccal mucosal epithelium, erythrocyte and leukocyte cells and chromosomal aberrations (CAs) in bone marrow cells, viability of liver and kidney cells were investigated. Four groups were randomly formed from male Swiss albino mice (one control and three treatment groups). The control group mice were provided tap water and the mice in the treatment groups were treated orally with three different doses of Paraquat (50, 100 and 200 mg/kg b.w) in the drinking water for 28 days. At the end of the application, all mice were sacrificed and routine preparation procedures were carried out to examine physiological, biochemical, oxidative stress and genetic parameters. Paraquat administration decreased physiological parameters (body, liver and kidney organ weights), and increased biochemical parameters (AST, ALT, BUN, creatinine and MDA). GSH levels were decreased depending on the dose. Kidney and liver damage were confirmed by the trypan blue test. Paraquat administration promoted MN formation in buccal mucosal epithelium, erythrocyte and leukocyte cells depending on the dose. The highest MN frequency was observed in leukocyte cells exposed to a dose of 200 mg/kg b.w of Paraquat. Deteriorations in DNA integrity as a result of MN formations were supported by the comet assay. In addition, Paraquat promoted CAs such as break, fragment, acentric, dicentric, gap and ring in bone marrow cells. Break damage was the most common among these damages. These observed genotoxic effects occured as a result of the interaction of DNA and DNA-related proteins with Paraquat. Molecular docking studies showed that Paraquat binds to histone H4 protein with high affinity and has a high intercalation potential. As a result, Paraquat herbicide caused a significant toxicity by changing physiological, biochemical, oxidative stress and genetic parameters of Swiss albino mice depending on the application dose.

## Introduction

Paraquat is a fast, effective and non-selective nitrogen herbicide, first synthesized in 1955. It is widely used as a drier and defoliant for the protection of agricultural products against weeds. Paraquat is rapidly absorbed by non-target organisms and is taken into the body by the mouth, respiration, skin and eyes. The toxic effects of Paraquat on plants are due to the production of free radicals after oxidation with oxygen molecules that cause photosynthesis damage. In animals, Paraquat is rapidly transported to all tissues and organs of the body. Paraquat cannot be metabolized in tissues. Instead, it is reduced to an unstable free radical that is reoxidized to form a superoxide anion and a cation. It is highly toxic to both humans and animals. It potentially causes acute respiratory distress syndrome. Severe Paraquat toxicity causes multi-organ failure with histological and functional changes in the lungs, kidneys, adrenal glands, liver and myocardium. Paraquat has a wide variety of mechanisms to induce cytotoxicity. Although the mechanisms of Paraquat are not fully understood, it is hypothesized that toxicity results from the generation of reactive oxygen species (ROS) via the redox cycle process and causes oxidative stress-related damage to cellular organelles, proteins, nucleic acids, and lipids^1–4^.

The aim of this study is to investigate the toxic effects of Paraquat on Swiss albino mice using physiological, biochemical, oxidative stress, and genetic parameters. While feed consumption, body and organ weight were examined as physiological parameters, serum ALT AST, BUN, creatinine and liver/kidney MDA and GSH levels were examined as biochemical parameters, and MN and CAs frequencies were examined as genetic parameters. Comet assay was applied to support the genotoxic effects of Paraquat and also the interaction of Paraquat with DNA and DNA-related proteins has been elucidated by molecular docking studies.

## Material and methods

### Experimental test material and animal care conditions

In this study, 24 male *Mus musculus* var. *albinos* (white laboratory mouse) were used as the experimental organism and Paraquat (Paraquat dichloride hydrate, CAS number: 50636-5 MG, Merck Company) was used as the chemical agent. Swiss albino mice were placed in stainless steel cages and maintained in a laboratory environment at 22 ± 3 °C and 55 ± 5% relative humidity, with 12 h of light and 12 h of dark cycles during the experimental period. All experiments were performed in accordance with the guidelines of the Animal Experiments Local Ethics Committee of Giresun University and approved by the Animal Ethics Committee of Giresun University (protocol number: 2010/01). This study was carried out in compliance with the ARRIVE guidelines.

### Experimental groups and experimental protocol

Mice were randomly divided into four (4) groups with six (6) animals in each group.

Group I: (Control);
Group II: 50 mg/kg b.w Paraquat;
Group III: 100 mg/kg b.w Paraquat;
Group IV: 200 mg/kg b.w Paraquat.

The animals in the control group were given tap water and the animals in groups II, III and IV were treated orally with 50, 100 and 200 mg/kg b.w of Paraquat in the drinking water for 28 days. The doses of Paraquat used in the study were determined by considering the dose ranges causing toxicity. Consumption of water, feed and Paraquat solutions were checked daily. One week before the experimental applications, the animals were brought to the laboratory where the experimental procedures would be carried out and their adaptation to the environment was ensured. At the end of 28 days, all animals were sacrificed under halothane anesthesia. During the experimental period, clinical signs and behaviors of all mice such as physical activity, diarrhea, tremor, paralysis, injury and death were monitored daily.

### Body and organ weight measurements

Mice were stunned with halothane administration, and their initial body weights were measured on the first day of the 28-day experimental period and final body weights at the end of the twenty-eighth day. After the mice were sacrificed at the end of the twenty-eighth day, liver and kidney weights were measured. Precision scales were used for body and organ weight measurements.

### Feed consumption

An albino mice consume about 3–4 g of feed per day^5^. Five grams of feed per mouse was added daily to each cage. The amount of feed remaining in the cages at the end of each week was removed and weighted, and feed consumption was calculated.

### Serum biochemical parameter measurements

Mice were anesthetized with halothane and blood samples (approximately 1.0 mL) were drawn from the heart into vacutainer tubes, centrifuged at 1.200×*g* for 10 min. at + 4 °C, and serum samples were stored in a deep-freeze at − 20 °C until analysis. AST/ALT enzyme activities and BUN/creatinine levels were measured on a Medispec 99M autoanalyzer using commercially available Teco Diagnostics (USA) kits^6^.

### MDA and GSH measurements

Mice were sacrificed by heart exsanguination under halothane anesthesia. The liver and kidneys of each mouse were removed, washed, dried under sterile conditions and prepared for biochemical measurements. Liver and kidney tissues were homogenized (Homogenizer, Ultraturrax Type T25-B, IKA Labortechnik, Germany) in 0.15 M cold KCl bath at 16.000 rpm for 3 min, the homogenates were centrifuged at 5.000×*g* at 4 °C for 1 h, the supernatants were collected and stored at − 40 °C in the deep-freeze until the measurements were made^7^. MDA and GSH levels of the tissues were measured with a UV mini-1240 (Shimadzu, Japan) brand spectrophotometer by fully applying the colorimetric method proposed by Yoshoiko et al.^8^ and Beutler^9^, respectively.

### Trypan blue test (TBT)

TBT was carried out according to the method described by Strober^10^. Cell suspensions of liver and kidney tissues were prepared according to the method suggested by Severgnini et al.^11^. Trypan blue (0.4% final concentration) and a liver or kidney cell suspension were mixed in a tube (1:1, 200 µL), the mixture was placed on slides and incubated for 3 min at room temperature. The number of dead (trypan blue-containing) cells was determined using light microscopy. A total of 600 cells were counted, 100 for each mouse in each group.

### MN test: buccal mucosal epithelium preparation

Mouths of anesthesized mice were washed with distilled water. Epithelial mucosa on the right and left sides of the mouth were scrapped using a slightly damp toothpick with a blunt tip, and epithelial cells were collected. Collected epithelial cells were transferred to sterile slides and the slides were left to dry for 15 min. Cells were then fixed in methanol (3 volumes) and acetic acid (1 volume) solution for 10 min, double stained with feulgen and fast green and covered with a coverslip with the help of entellan^12^.

### MN test: polychromatic erythrocyte preparation

Mouse erythrocyte MN testing was performed according to the method suggested by Te-Hsiu et al.^13^. Mice were stunned by halothane application, blood samples were collected from tail veins. Blood (5 µL) was mixed with 3% EDTA solution and spread on sterile slides. Cells on the slide were fixed in 70% ethanol for 2 min. and left to dry for one day at room temperature. Slides were then stained with giemsa (*5%*) for 15 min, and covered with a coverslip using entellan.

### MN test: leukocyte preparation

Mice were anesthetized with halothane and blood samples were collected. The samples were centrifuged at 5000 rpm for 10 min., the upper clear part was discarded, 5 mL of 0.075 M KCl solution was added to the remaining residue, and left at room temperature for 20 min. The tubes were then centrifuged again at 5000 rpm for 10 min. The upper part was removed, and 5 mL of the washing solution consisting of a mixture of methanol (3 volumes) and glacial acetic acid (1 volume) was added to the sample remaining in the tube. Tubes were held at − 20 °C for 30 min. Leukocytes were then spread on sterile slides, stained with giemsa (*5%*) and covered with a coverslip with entellan^14^.

### MN determinations

The presence of MN in all three cell types (buccal mucosal epithelium, erythrocyte and leukocytes) was determined according to the criteria described by Fenech et al.^15^. According to this:

- The size of the MN should be about 1/3 of the cell nucleus.
- MN and cell nucleus should have a similar color when stained.
- When the membranes of the MN and the cell nucleus come into contact, the boundary between them should be clearly discernible.

1000 cells were counted in each group to detect MN formation in all three cell types. MN photographed at × 500 magnification under Irmeco IM-450 TI model research microscope.

### Determination of CAs

Colchicine (0.025%) was given to mice intraperitoneally 2 h before sacrificing under halothane anesthesia. Bone marrow cells from mouse femurs were aspirated, washed in saline, treated with 0.075 M KCl, fixed in Carnoy’s fixative and stained with giemsa (5%)^16^. CAs were detected using an Irmeco IM-450 TI model research microscope and classified according to the criteria proposed by Savage^17^.

### Comet assay (single-cell gel electrophoresis)

The protocol of Tice et al.^18^ was used for alkaline single cell gel electrophoresis with slight modifications. Slides were dipped in 1% normal melting point agarose for coating and allowed to dry at 37 °C. 10 µL of blood were added to 120 µL of 0.5% low‐melting point agarose at 37 °C, layered onto a coated slide, covered with a coverslip and left at 4 °C for 5 min to solidify the agarose. The coverslip was removed and the slides were immersed into a lysis solution (2.5 M NaCl, 100 mm Na_2_EDTA, 10 mM Tris‐HCl buffer, pH 10, 1% Triton X‐100) for approximately 1 h. After lysis, the slides were transferred to a horizontal gel electrophoresis tank with a fresh and cooled alkaline buffer. After a 20‐min. DNA unwinding period, electrophoresed at 0.86 V/cm (20 V, 300 mA) for 20 min. Slides were stained using ethidium bromide staining solution after carefully flushing three times with tris-buffer (0.4 M Tris, pH 7.5) for 5 min. The preparations were washed with cold water to remove excess stain and covered with a coverslip. To prevent DNA damage, all steps were performed in low light and analyzed by fluorescence microscopy. Cells appearing as comets were evaluated by Comet Assay software version 1.2.3b^19^ with the parameters of tail DNA percentage, tail moment, and olive tail moment. Two hundred cells were evaluated for each treatment group.

### Molecular docking

Molecular docking was performed to analyze potential interactions of Paraquat with histone and DNA molecules. The cyro-em 3D structure of tubulin (alpha-1B chain and tubulin beta chain) (6RZB)^20^, the crystal 3D structure of histone proteins (histone H3.1, histone H4, histone H2A and histone H2B type 1-A) (3X1T)^21^ and the 3D structures of B-DNA dodecamer (PDB ID: 1bna)^22^, B-DNA dodecamer d (PDB ID: 195d)^23^ and DNA (PDB ID: 1cp8)^24^ molecules were obtained from the protein data bank. The 3D structure of Paraquat (PubChem CID: 15939) was retrieved from the PubChem. Proteins and DNA molecules were prepared using Biovia Discovery Studio 2020 Client for docking. During the preparation process, the active sites were determined, water molecules and ligands were removed and polar hydrogen atoms were added. Energy minimization of proteins was done with Gromos 43B1 using Swiss-PdbViewer^25^ (v.4.1.0) software whereas energy minimization of the 3D structure of Paraquat was accomplished with the uff-force field employing Open Babel v.2.4.0 software^26^. The molecular docking process was carried out with the grid box containing the active sites of proteins and the entire structure of DNA. Then docking was performed using Autodock 4.2.6 software^27^ based on Lamarckian genetic algorithm. The docking analysis and 3D visualizations were performed with Biovia Discovery Studio 2020 Client.

### Statistical analysis of data

Statistical analysis was carried out using SPSS for Windows V 22.0 (SPSS Inc, Chicago, IL, USA) package program. One-way ANOVA and Duncan’s tests were applied to evaluate statistical differences between the groups. Data are presented as mean ± SD and were considered statistically significant when the p value was < 0.05.

## Result and discussion

### Analysis of physiological parameters

The effects of Paraquat on selected physiological parameters in Swiss albino mice are shown in Table 1. The highest body weight and liver and kidney organ weight values were measured in Group I (control). In the groups administered with Paraquat, the body and organ weight values decreased considerably depending on the dose of Paraquat, and this decrease was statistically significant (p < 0.05). The greatest decrease in body and organ weight values was measured in mice treated with 200 mg/kg b.w of Paraquat. When compared to the control group, body weight decreased approximately 9 times, liver organ weight approximately 1.8 times and kidney organ weight approximately 1.9 times in Group IV. In the literature, there are some studies with different organisms that support our results. Reddy et al.^4^ reported a statistically significant decrease in body weights of male albino Wistar rats administered Paraquat at a dose of 40 mg/kg b.w daily for 21 days compared to the control group. Riahi et al.^28^ observed that Paraquat had no significant effect on body weight and liver, kidney and spleen organ weights of the mice at dosages (≤ 1.0 mg/kg b.w) Paraquat during twenty-one days. Hassan et al.^29^ reported that body weight decreased on the seventh day of Paraquat administration, and liver, kidney, heart and lung organ weights on the first three days in rabbits exposed intraperitoneally to 3, 6, and 12 mg/kg b.w doses of Paraquat.

**Table 1:** Effects of Paraquat on feed consumption, body and organ weights.

Parameters	Group I	Group II	Group III	Group IV
Weight gain (g)	+ 10.16^a^	+ 7.51^b^	+ 4.28^c^	+ 1.13^d^
Initial body weight	34.70 ± 1.85	34.64 ± 1.82	34.50 ± 1.80	34.75 ± 1.86
Final body weight	44.86 ± 2.12	42.15 ± 1.86	38.78 ± 1.64	35.88 ± 1.57
Liver weight (g)	2.38 ± 0.32^a^	1.98 ± 0.27^b^	1.70 ± 0.21^c^	1.35 ± 0.18^d^
Kidney weight (g)	1.55 ± 0.28^a^	1.36 ± 0.22^b^	1.10 ± 0.18^c^	0.83 ± 0.15^d^
F.C. 7th day (g)	150.0	145.8	142.7	139.5
F.C. 14th day (g)	154.4	142.5	133.6	124.3
F.C. 21st day (g)	158.6	135.6	125.8	105.7
F.C. 28th day (g)	163.8	130.3	110.5	81.9

Group I: Control, Group II: 50 mg/kg b.w Paraquat, Group III: 100 mg/kg b.w Paraquat, Group IV: 200 mg/kg b.w Paraquat.

*F.C* Feed consumption over the previous 7 days.

Values are shown as mean ± SD (n = 6). Means shown with different letters on the same line are significantly different (p < 0.05).

Food consumption decreased with increasing Paraquat exposure dose (Table 1) which may have been responsible for the decrease in body weight gain and organ weight. Reddy et al.^4^ stated that the main reason for the weight loss observed in male Wistar rats treated with 40 mg/kg b.w Paraquat was reduced feed and water intake. Lalruatfela et al.^30^ reported that diarrhea and anorexia developed in the second week of administration in female Wistar rats exposed to Paraquat for 28 days. Similarly, Haripriya et al.^3^ attributed the decrease in body weight of Wistar albino rats administered 24 mg/kg b.w dose of Paraquat to the decrease in feed and water intake by the animals.

### Biochemical analysis

AST, ALT, BUN, and creatinine levels were significantly elevated at all Paraquat treatment levels in a dose-dependent manner (Table 2). Lalruatfela et al.^30^ reported statistically significant increases in AST, ALT and creatinine levels in female Wistar rats exposed to Paraquat at doses of 10, 15, 25 mg/kg b.w by oral gavage for 28 days. Haripriya et al.^3^ determined that acute Paraquat administration at a dose of 24 mg/kg b.w for 24, 48 and 72 h increased serum AST and ALT enzyme activity and creatinine levels in Wistar albino rats at all three time periods. Especially increases in biochemical parameters indicate kidney and liver damage and these damages were also confirmed by trypan blue test (TBT). No dead cells were found in the liver and kidney of the control group using the TBT test. Paraquat exposure caused a significant dose-dependent increase in dead cells in both the liver and kidney (Table 2). Our results are supported by the results of histopathological studies carried out by other researchers on the effects of Paraquat on liver and kidney tissues. For example, Lalruatfela et al.^30^ reported that there were granular and vacuolar changes in the lungs and livers of female Wistar rats exposed to Paraquat, as well as congestion, emphysema, congestion and edema in the lungs, and atrophy and hypertrophy in the kidneys. They concluded that Paraquat caused histopathological changes in the liver and kidneys of rats, resulting in hepatotoxic and nephrotoxic effects. Similarly, Haripriya et al.^3^ showed severe damages formed in liver parenchyma, hepatocyte cells, and kidneys as the main reason for the increase in AST and ALT enzyme activity and creatinine levels as a result of acute administration of Paraquat at a dose of 24 mg/kg b.w.

**Table 2:** Effects of Paraquat on selected biochemical parameters and viability of liver and kidney cells.

Parameters	Group I	Group II	Group III	Group IV
AST (U/L)	150.00 ± 5.36^d^	174.00 ± 6.85^c^	196.00 ± 7.58^b^	231.00 ± 8.52^a^
ALT (U/L)	100.00 ± 4.96^d^	121.00 ± 5.19^c^	140.00 ± 5.88^b^	168.00 ± 7.74^a^
BUN (mg/L)	310.00 ± 9.24^d^	348.00 ± 10.73^c^	382.00 ± 11.82^b^	421.00 ± 13.64^a^
Creatinine (mg/L)	7.52 ± 1.98^d^	9.38 ± 2.15^c^	13.40 ± 2.85^b^	17.54 ± 3.16^a^
MDA_Liver_ (nmol/g)	0.346 ± 0.34^d^	0.391 ± 0.44^c^	0.462 ± 0.53^b^	0.540 ± 0.62^a^
MDA_Kidney_ (nmol/g)	0.285 ± 0.16^d^	0.350 ± 0.23^c^	0.548 ± 0.42^b^	0.764 ± 0.54^a^
GSH_Liver_ (mg/g)	0.412 ± 0.86^a^	0.386 ± 0.68^b^	0.350 ± 0.55^c^	0.316 ± 0.42^d^
GSH_Kidney_ (mg/g)	0.360 ± 0.66^a^	0.298 ± 0.51^b^	0.216 ± 0.38^c^	0.154 ± 0.15^d^
Liver cell death	0.00 ± 0.00^d^	65.0 ± 3.36^c^	102 ± 5.44^b^	154 ± 6.13^a^
Kidney cell death	0.00 ± 0.00^d^	47 ± 2.44^c^	88 ± 4.35^b^	128 ± 4.98^a^

Group I: Control, Group II: 50 mg/kg b.w Paraquat, Group III: 100 mg/kg b.w Paraquat, Group IV: 200 mg/kg b.w Paraquat.

*AST* aspartate aminotransferase, *ALT* alanine aminotransferase, *BUN* blood urea nitrogen, *MDA* malondialdehyde, *GSH* glutathione.

In cell viability test, 100 cells were counted for each mouse in each group, a total of 600 cells. Values are shown as mean ± SD (n = 6). Means shown with different letters on the same line are significantly different (p < 0.05).

### Oxidative stress analysis

The effects of Paraquat on selected oxidative stress parameters in Swiss albino mice are shown in Table 2. Paraquat exposure caused a significant dose-dependent increase in MDA levels and a decrease in GSH levels in liver and kidney tissues. Ray et al.^31^ reported that administration of 1.5 and 7.5 mg/kg b.w of Paraquat for 3 and 7 days in rats caused an increase in MDA levels and a decrease in GSH levels in blood cells, liver, kidney and lung tissues, depending on the application time and dose. Mirzaee et al.^32^ reported that administration of 30 mg/kg b.w Paraquat intraperitoneally to albino mice caused an increase in lung MDA levels and a decrease in GSH levels by increasing oxidative stress. El-Boghdady et al.^33^ showed that exposure to 50 mg/kg b.w of Paraquat intraperitoneally in albino mice caused liver damage, resulting in an increase in MDA levels and a decrease in GSH levels. GSH is a powerful antioxidant produced in the liver. It plays a key role in the detoxification process that occurs in the liver^34^. MDA is the end product of the lipid peroxidation process. The decrease in GSH and increase in MDA is indicative of oxidative stress elicited at all Paraquat treatment levels.

### Genotoxic analysis

**Figure 1:**
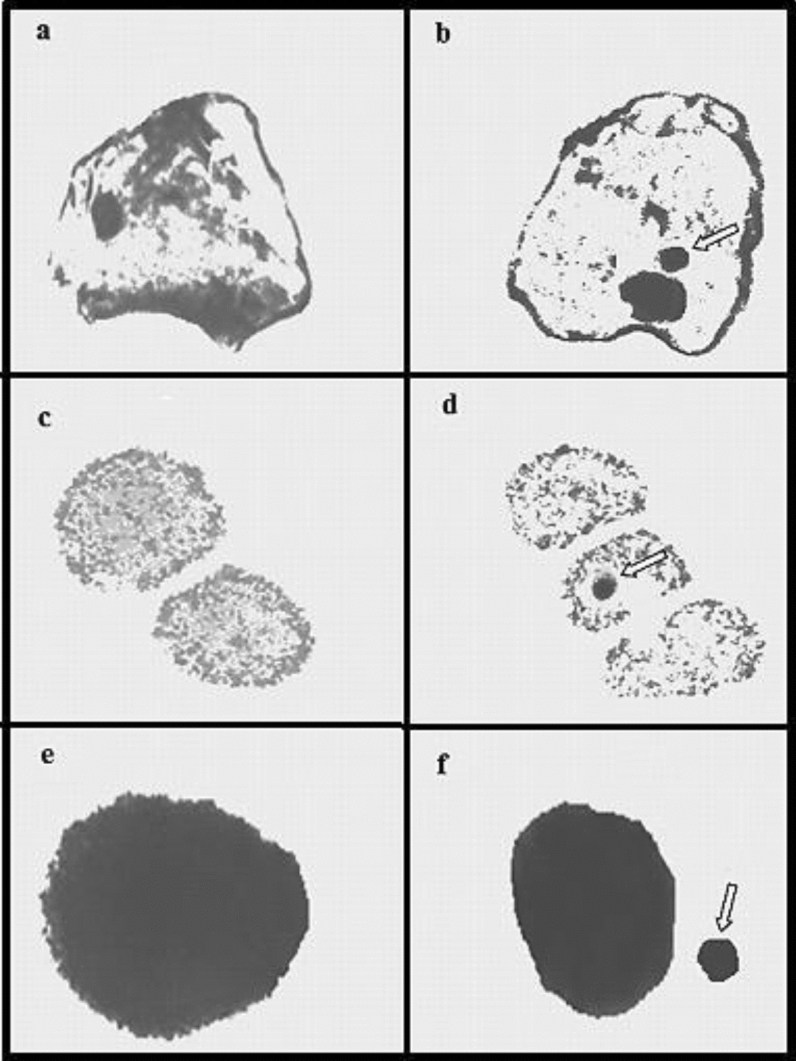
MN formation induced by Paraquat in different cells. Buccal mucosal epithelium normal appearance (**a**), buccal mucosal epithelium with MN (**b**), erythrocyte cell normal appearance (**c**), erythrocyte cell with MN (**d**), leukocyte cell normal appearance-*lymphocyte* (**e**), leukocyte cell with MN-*lymphocyte* (**f**).

Paraquat caused a significant dose-dependent increase in MN formation in buccal mucosal epithelium, erythrocyte and leukocyte cells; along with various CAs in bone marrow cells (Table 3, Fig. 1). Melchiorri et al.^35^ reported statistically significant increases in the MN numbers of normochromatic and polychromatic erythrocyte cells of albino mice administered Paraquat at a dose of 15 mg/kg b.w intraperitoneally. Rios et al.^36^ reported that 1.5, 3, 5, 7, 15 and 23 mg/kg Paraquat doses induced CAs such as gap, breaks and fragments in bone marrow and spermatozoa cells of albino mice, and the rate of these damages increased depending on the application dose. Gateva and Kulekova^37^ observed that doses of Paraquat from 10^–4^ to 10^–6^ caused dose-dependent apoptosis and CAs in human lymphocyte cells.

**Table 3:** Genotoxic effects of Paraquat.

Parameters	Group I	Group II	Group III	Group IV
BME MN	0.00 ± 0.00^d^	14.32 ± 1.85^c^	29.61 ± 2.66^b^	40.48 ± 3.50^a^
Erythrocyte MN	0.00 ± 0.00^d^	24.66 ± 2.37^c^	46.83 ± 4.26^b^	68.59 ± 6.74^a^
Leukocyte MN	0.16 ± 0.98^d^	30.95 ± 2.94^c^	66.72 ± 5.78^b^	92.56 ± 8.74^a^
CAs breaks	0.00 ± 0.00^d^	21.96 ± 2.38^c^	42.85 ± 3.94^b^	60.35 ± 5.78^a^
Fragments	0.00 ± 0.00^d^	16.76 ± 1.58^c^	28.50 ± 2.78^b^	43.51 ± 4.16^a^
Acentrics	0.00 ± 0.00^d^	9.84 ± 1.16^c^	19.75 ± 2.14^b^	28.44 ± 3.10^a^
Dicentrics	0.00 ± 0.00^d^	6.30 ± 1.10^c^	15.72 ± 1.93^b^	21.36 ± 2.85^a^
Gaps	0.00 ± 0.00^d^	4.85 ± 0.96^c^	10.50 ± 1.75^b^	17.93 ± 2.24^a^
Rings	0.00 ± 0.00^d^	2.71 ± 0.88^c^	8.86 ± 1.58^b^	15.30 ± 1.95^a^

Group I: Control, Group II: 50 mg/kg b.w Paraquat, Group III: 100 mg/kg b.w Paraquat, Group IV: 200 mg/kg b.w Paraquat.

*MN* micronucleus, *BME* buccal mucosal epithelium.

Values are shown as mean ± SD (n = 6). For the frequency of MN 1.000 cells in each group and 600 cells for CAs were analyzed. Means shown with different letters on the same line are significantly different (p < 0.05).

### Comet assay

**Figure 2:**
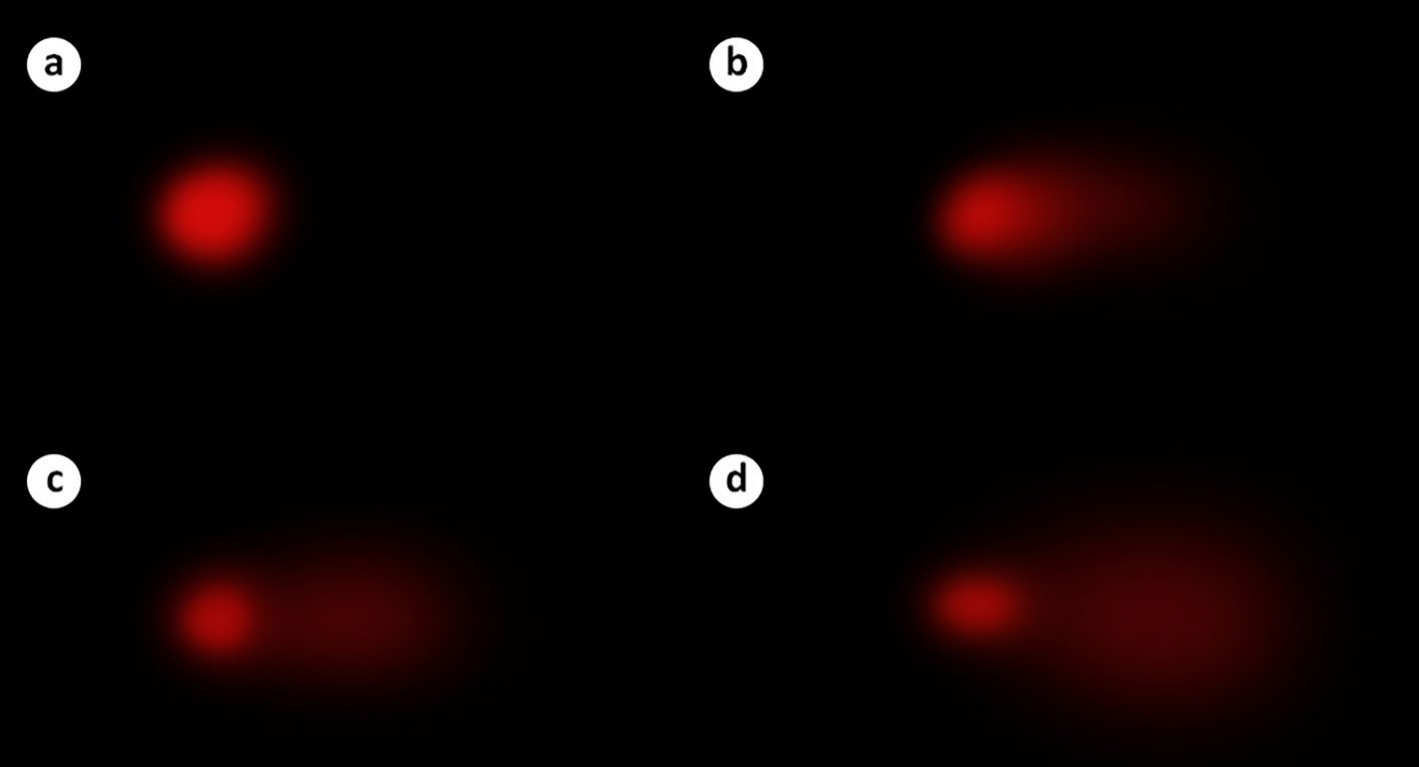
Comet assay analysis in leukocyte cell nuclei of Paraquat-treated mice. Control (**a**), 50 mg/kg b.w. Paraquat (**b**), 100 mg/kg b.w. Paraquat (**c**), 200 mg/kg b.w. Paraquat (**d**).

DNA damage caused by Paraquat in leukocyte cell nuclei of swiss albino mice was evaluated using single-cell gel electrophoresis with the percentage of tail DNA, tail moment and olive tail moment. The percentage of head DNA was significantly decreased in a Paraquat dose-dependent manner; while, the percentage of tail DNA, tail moment, and olive tail moment increased significantly with increasing dose (Fig. 2, Table 4). These results, in combination with results of the MN and CA evaluations indicate that Paraquat disrupts DNA integrity.

**Table 4:** Detection of DNA damage caused by Paraquat in leukocyte cells of Swiss albino mice.

Groups	Head DNA (%)	Tail DNA (%)	Tail moment	Olive tail moment
I	98.78 ± 0.23^d^	1.22 ± 0.23^d^	0.16 ± 0.02^d^	0.82 ± 0.18^d^
II	58.84 ± 1.74^c^	41.16 ± 1.74^c^	48.28 ± 1.12^c^	24.16 ± 1.76^c^
III	46.96 ± 1.59^b^	53.04 ± 1.59^b^	65.56 ± 1.46^b^	42.82 ± 1.34^b^
IV	28.02 ± 1.33^a^	71.98 ± 1.33^a^	107.16 ± 1.72^a^	72.58 ± 1.18^a^

Group I: Control, Group II: 50 mg/kg b.w Paraquat, Group III: 100 mg/kg b.w Paraquat, Group IV: 200 mg/kg b.w Paraquat.

Values are shown as mean ± SEM. Means shown with different letters on the same column are significantly different (p < 0.05).

### Molecular docking

**Figure 3:**
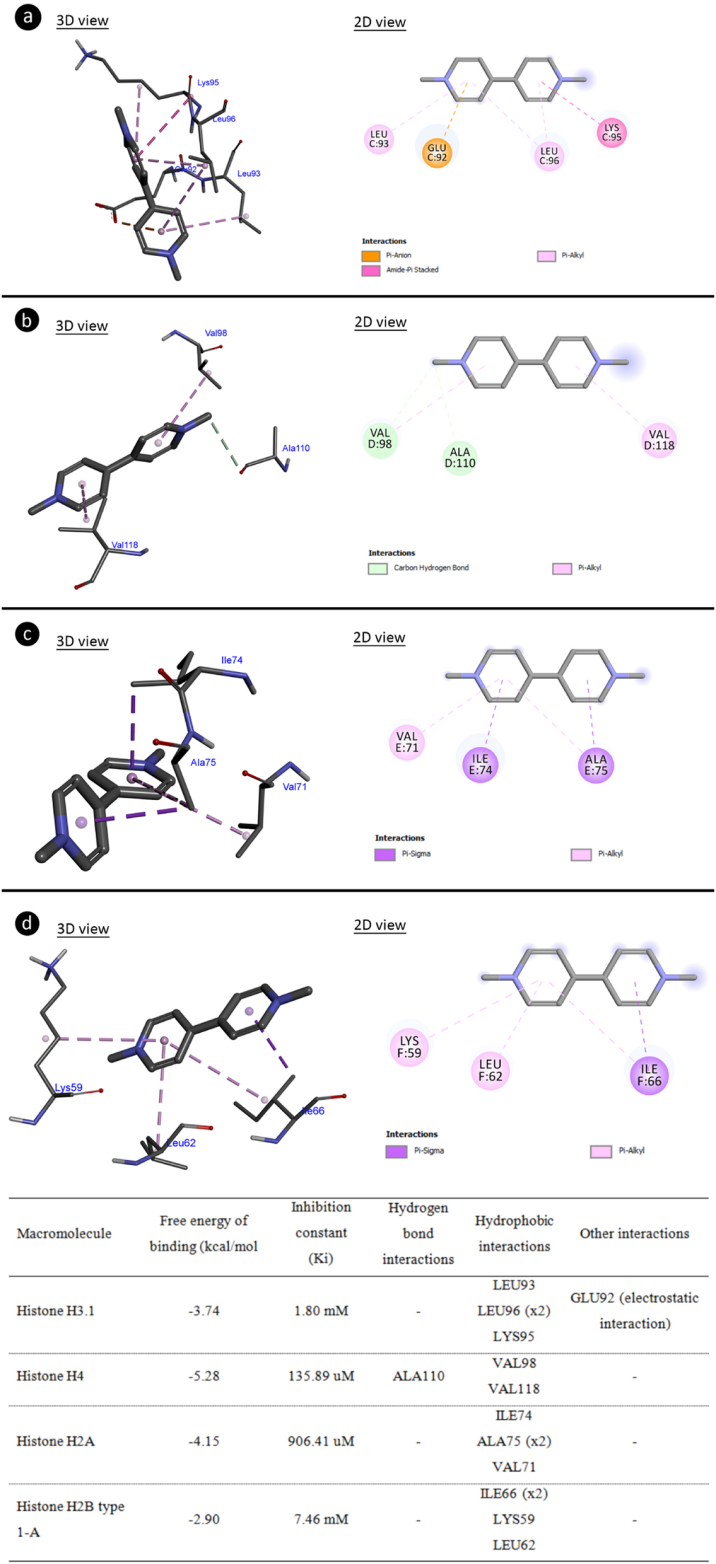
The structural interaction of Paraquat with histone proteins. Histone H3.1-Paraquat complex (**a**), histone H4-Paraquat complex (**b**), histone H2A-Paraquat complex (**c**), histone H2B type 1A-Paraquat complex (**d**) and the binding affinities/interactions of Paraquat with histone proteins.

Molecular docking studies, based on binding energy, revealed that Paraquat is capable of interacting with histone proteins (Fig. 3). Paraquat exhibited the strongest binding affinity to histone H4 protein with − 5.28 kcal/mol. Hydrogen bonding interactions between Paraquat and histone H3.1 protein did not occur, but hydrophobic interactions with Leu93, Leu96 and Lys95 residues and electrostatic interactions with Glu92 occurred with a binding energy of − 3.74 kcal/mol and an inhibition constant of 1.80 mM. The Ala110 residue of histone H4 protein can interact with Paraquat through hydrogen bonding, and Val98 and Val118 residues can elicit hydrophobic interactions with a binding energy − 5.28 kcal/mol and inhibition constant 135.89 uM. There was no hydrogen bonding contacts between Paraquat and histone H2A protein; however, there were hydrophobic interactions with Ile74, Ala75 and Val71 residues, with a binding energy of − 4.15 kcal/mol and an inhibition constant of 906.41 uM. Paraquat showed no hydrogen bond interactions with histone H2B protein, but hydrophobic interactions with residues Ile66, Lys59 and Leu62 with a binding energy of − 2.90 kcal/mol and an inhibition constant of 7.46 mM. Paraquat had the highest binding affinity to histone H4 among histone proteins. H4 is involved in the organization of ‘beads on string’ in the nucleosome structure. This situation has an important place in the packaging of DNA, the formation of chromosomes and the stability of the genome. The high binding of Paraquat to the H4 protein can cause a deterioration in genome stability. Particularly, the DNA integrity disruption observed in the comet test may be associated with the Paraquat-histone H4 interaction.

**Figure 4:**
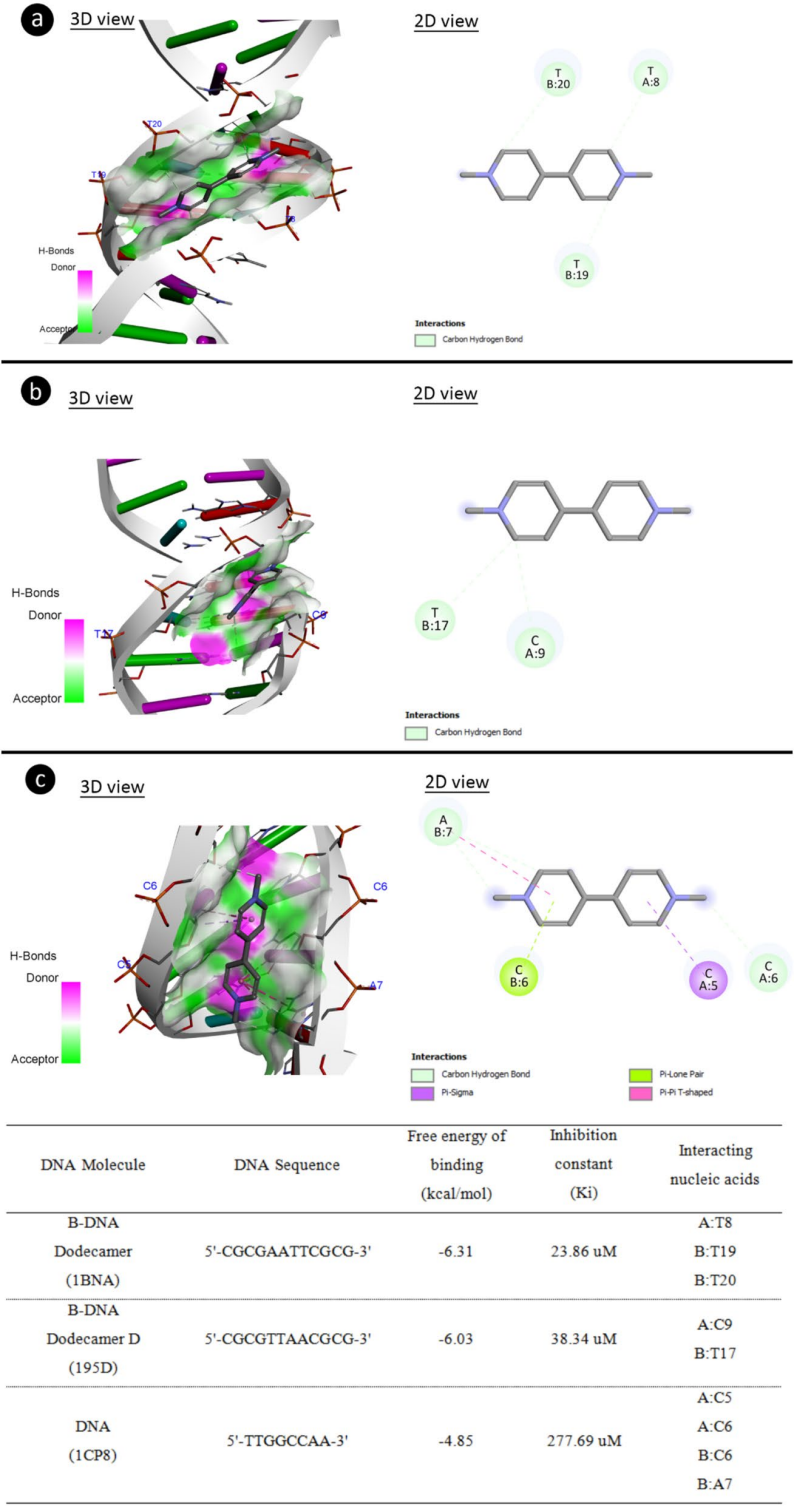
The structural interaction of Paraquat with DNA molecules. 1BNA (**a**), 195D (**b**), 1CP8 (**c**).

Possible interactions of Paraquat with DNA molecules were also investigated (Fig. 4). Paraquat had a contact with B-DNA dodecamer (1BNA) with − 6.31 kcal/mol binding energy and an inhibition constant of 23.86 uM. Paraquat showed a carbon hydrogen bond interaction with T8 base in the A chain and with T19 and T20 bases in the B chain. Paraquat can form a binds with carbon-hydrogen bond to bases C9 (chain A) and T17 (chain B) of B-DNA Dodecamer D (195D) with binding energy − 6.03 kcal/mol and inhibiting constant of 38.34 uM. The C5–C6 (chain A) and C6-A7 (chain B) bases of DNA (1CP8) may complex with Paraquat with a binding energy − 4.85 kcal/mol and an inhibition constant of 277.69 uM. The results of molecular docking studies performed with Paraquat and different DNA molecules revealed that Paraquat has the ability to interact with the same and different strands in DNA molecules and intercalation potential. Intercalation occurs by stacking chemicals between adjacent base pairs in DNA without forming any covalent bonds between the chemical and DNA^38^. Such DNA intercalators are not DNA adductors, but the intercalation of chemicals may be promutagenic. Intercalation also causes the supercoiled DNA to unravel, which can ultimately prevent DNA from being recognized by DNA-binding proteins and other regulatory factors^39^. Intercalators agents such as Paraquat have diverse and multiple biological effects on DNA. Inhibition of RNA and DNA synthesis, frameshift mutations, and protein-associated DNA breaks are some of these effects^40^. Especially DNA breaks lead to high CAs and MN formations. The high frequency of MN and CAs detected in the Paraquat group in this study shows that Paraquat causes protein-associated DNA breaks. Molecular docking analysis with histone proteins also confirms this finding.

## Conclusion

Paraquat exposure at dosages ranging from 50 to 200 mg/kg b.w. elicited toxicity as evidenced by changes in the physiological, biochemical and genetic structure of Swiss albino mice. The results we obtained are also in line with previous studies studies. However, our study is the most comprehensive study that deals with the effects of Paraquat in terms of physiological, biochemical and genetic aspects. It is also the first study to investigate MN formation caused by Paraquat genotoxicity in buccal mucosal epithelium and leukocyte cells. The genotoxicity mechanism of Paraquat was investigated by molecular docking studies and it was determined that Paraquat was an intercalator, causing deterioration in DNA integrity and protein-associated DNA breaks. In addition, paraquat caused damage to liver and kidney cell membranes, resulting in an increase in MDA levels, caused a decrease in glutathione level by promoting the formation of free radicals and induced oxidative stress. The present study provides a comprehensive assessmet of the mechanisms by which Paraquat elicits toxicity.

## References

[1] Chohan MS, Tahir M, Lone KP, Sami W, Munir B (2010). Paraquat induced hepatotoxicity in albino mice. Pak. J. Zool..

[2] Ranjbar A (2014). Evidence of oxidative damage in Paraquat toxicity. Zahedan J. Res. Med. Sci..

[3] Haripriya B, Lakshman M, Sudha V (2017). Influence of Paraquat induced acute toxicity on body weights and haemoto-biochemical parameters in experimental. Int. J. Sci. Res..

[4] Reddy KBAK, Jeevanalatha M, Lakshman M, Rani MU (2019). The toxic effects of Paraquat on body weights and haematological parameters in male albino wistar rats and its amelioration with vitamin C. Int. J. Curr. Microbiol. Appl. Sci..

[5] Saruhan BG, Dereli S (2016). Reproduction, shelter and feeding of the experimental animals. Dicle Univ. J. Vet. Med..

[6] Cavusoglu K, Yapar K, Yalcın E, Oruc E (2010). Protective effect of royal Jelly on some biochemical parameters in cadmium-treated albino mice. Fresenius Environ. Bull..

[7] Yalcin E, Oruc E, Cavusoglu K, Yapar K (2010). Protective effect of grape seed extract on doxorubicin-induced nephrotoxicity and hepatotoxicity in albino mice. Fresenius Environ. Bull..

[8] Yoshioka T, Kawada K, Shimada T, Mori M (1979). Lipid peroxidation in maternal and cord blood and protective mechanism against activated-oxygen toxicity in the blood. Am. J. Obstet. Gynecol..

[9] Beutler E (1963). Improved method for the determination of blood glutathione. J. Lab. Clin. Med..

[10] Strober W (2015). Trypan blue exclusion test of cell viability. Curr. Protoc. Immunol..

[11] Severgnini M (2012). A rapid two-step method for isolation of functional primary mouse hepatocytes: Cell characterization and asialoglycoprotein receptor based assay development. Cytotechnology.

[12] Taşlı B, Çiçek F, Yalçın E, Demirtaş G, Çavuşoğlu K (2015). The protective effect of green tea extract on formaldehyde toxicity: Genotoxic evaluation in Swiss albino mice. Cumhuriyet Sci. J..

[13] Te-Hsiu MA, Zhou X, Loarco GF, Arreola GG, Lecona SU (1995). Mouse-erythrocyte micronucleus (MUS-EMN) assay on the clastogenicity of industrial wastewater. Rev. Int. Contam. Ambient..

[14] Acar, A., Yalçın, E., Yapar, K. & Çavuşoğlu, K. Protective effect of royal jelly against changes in physiological and genetic structure promoted by lambda cyhalothrin. In *Black Sea 1st International Multidisciplinary Scientific Studies Congress*, 463–469 (2019).

[15] Fenech M, Chang WP, Kirsch-Volders M, Holland N, Bonassi S, Zeiger E (2003). HUMN project: Detailed description of the scoring criteria for the cytokinesis-block micronucleus assay using isolated human lymphocyte cultures. Mutat. Res. Genet. Toxicol. Environ. Mutagen..

[16] Beyersmann D, Hechtenberg S (1997). Cadmium, gene regulation, and cellular signaling in mammalian cells. Toxicol. Appl. Pharmacol..

[17] Savage JR (1976). Classification and relationships of induced chromosomal structural changes. J. Med. Genet..

[18] Tice RR (2000). Single cell gel/comet assay: Guidelines for in vitro and in vivo genetic toxicology testing. Environ. Mol. Mutagen..

[19] Końca K (2003). A cross-platform public domain PC image-analysis program for the comet assay. Mutat. Res..

[20] Lacey SE, He S, Scheres SH, Carter AP (2019). Cryo-EM of dynein microtubule-binding domains shows how an axonemal dynein distorts the microtubule. Elife.

[21] Padavattan S (2015). Structural and functional analyses of nucleosome complexes with mouse histone variants TH2a and TH2b, involved in reprogramming. Biochem. Biophys. Res. Commun..

[22] Drew HR (1981). Structure of a B-DNA dodecamer: Conformation and dynamics. Proc. Natl. Acad. Sci..

[23] Balendiran K, Rao ST, Sekharudu CY, Zon G, Sundaralingam M (1995). X-ray structures of the B-DNA dodecamer d (CGCGTTAACGCG) with an inverted central tetranucleotide and its netropsin complex. Acta Crystallogr. D Biol. Crystallogr..

[24] Katahira R (1998). Solution structure of the novel antitumor drug UCH9 complexed with d (TTGGCCAA) 2 as determined by NMR. Nucleic Acids Res..

[25] Guex N, Peitsch MC (2005). SWISS-MODEL and the Swiss-Pdb Viewer: An environment for comparative protein modeling. Electrophoresis.

[26] O'Boyle NM (2011). Open Babel: An open chemical toolbox. J. Cheminform..

[27] Morris GM (2009). AutoDock4 and AutoDockTools4: Automated docking with selective receptor flexibility. J. Comput. Chem..

[28] Riahi B (2010). Immunotoxicity of Paraquat after subacute exposure to mice. Food Chem. Toxicol..

[29] Hassan RA, Afzal M, Ali M, Gubler CJ (1989). Effect of Paraquat administered intraperitoneally on the nonpolar lipids of rabbits. Ecotoxicol. Environ. Saf..

[30] Lalruatfela PL, Saminathan M, Ingole RS, Dhama K, Joshi MV (2014). Toxicopathology of Paraquat herbicide in female Wistar rats. Asian J. Anim. Vet. Adv..

[31] Ray S, Sengupta A, Ray A (2007). Effects of Paraquat on anti-oxidant system in rats. Indian J. Exp. Biol..

[32] Mirzaee S, Mansouri E, Shirani M, Zeinvand-Lorestani M, Khodayar MJ (2019). Diosmin ameliorative effects on oxidative stress and fibrosis in Paraquat-induced lung injury in mice. Environ. Sci. Pollut. Res..

[33] El-Boghdady NA, Abdeltawab NF, Nooh MM (2017). Resveratrol and montelukast alleviate Paraquat-induced hepatic injury in mice: Modulation of oxidative stress, inflammation, and apoptosis. Oxid. Med. Cell. Longev..

[34] Pizzorno J (2014). Glutathione. Integr. Med..

[35] Melchiorri D (1998). Melatonin reduces Paraquat-induced genotoxicity in mice. Toxicol. Lett..

[36] Rios AC, Salvadori DM, Oliveira SV, Ribeiro LR (1995). The action of the herbicide Paraquat on somatic and germ cells of mice. Mutat. Res. Fund. Mol. Mech. Mutag..

[37] Gateva S, Kulekova S (2008). Chromosome aberrations and apoptosis induced by Paraquat corresponding with cell cycle delay in human lymphocytes in vitro. J. Environ. Prot. Ecol..

[38] Ferguson LR, Denny WA (2007). Genotoxicity of non-covalent interactions: DNA intercalators. Mutat. Res..

[39] Preston RJ, Ross JA, McQueen CA (2018). Mechanisms: DNA-reactive agents. Comprehensive Toxicology.

[40] Filipski J (1983). Competitive inhibition of nicking—Closing enzymes may explain some biological effects of DNA intercalators. FEBS Lett..

